# ‘When They Struggle, I Cannot Sleep Well Either’: Perceptions and Interactions Surrounding University Student and Teacher Well-Being

**DOI:** 10.3389/fpsyg.2020.578378

**Published:** 2020-09-09

**Authors:** Lisa Kiltz, Raven Rinas, Martin Daumiller, Marjon Fokkens-Bruinsma, Ellen P. W. A. Jansen

**Affiliations:** ^1^Department of Teacher Education, University of Groningen, Groningen, Netherlands; ^2^Department of Psychology, University of Augsburg, Augsburg, Germany

**Keywords:** higher education, well-being, student-teacher interaction, positive psychology, SDT, resilience, university

## Abstract

A wealth of evidence has indicated that both students and teachers experience high levels of stress, burnout, and ultimately compromised well-being in the university context. Although numerous studies have investigated well-being among university students, and other studies have addressed well-being among university teachers, these lines of research are often conducted in isolation from one another. This is surprising, as the importance of considering reciprocal links between students and teachers has been suggested in several empirical studies. Additionally, when researching well-being in academia, the conceptualizations tend to differ from study-to-study. The present research therefore investigated how students and teachers conceptualize well-being at the university based on their personal experiences, as well as how student and teacher well-being interact. To examine this, six university students (50% female), and ten teachers (50% female) from Germany and the Netherlands participated in semi-structured interviews. Qualitative analysis using a multistage coding process revealed detailed insights concerning students’ and teachers’ perceptions of well-being that coincided with positive psychology, resilience, multifaceted, and basic psychological need fulfillment approaches. Moreover, an interaction between students’ and teachers’ well-being became apparent, including several factors such as the student-teacher relationship that in turn, contributed to both population’s well-being. The present findings lend evidence toward a more coherent conceptualization of well-being and are discussed in terms of suggestions for initiatives that simultaneously support both populations, for example, through the student-teacher relationship.

## Introduction

*“I used to think that I had a lot of impact on students and student well-being and I could really stress myself about students who were [stressed out]. Like- yeah REALLY. So, I was the one laying, late at night, thinking about how should we do this, and how can we make it to the deadline. And, and um (p), so tried very, various things, and I now end up thinking my, I think my role is rather limited. More limited than I originally thought.”* (university teacher)

For university students and teachers alike, academic environments can be stimulating, informative, and socially enriching; however, they can also be competitive and stressful. Therefore, the pursuit of success in academia constitutes a challenging process, making students and teachers susceptible to compromised well-being (Henning et al., 2018). In line with this, it is known that university students experience high levels of academic distress and mental health problems. These include psychological and emotional distress (Larcombe et al., 2015; Deasy et al., 2016; Baik et al., 2017), burnout (de Broer, 2017), and elevated prevalence of depression, anxiety, and stress disorders (Wong et al., 2006; Bayram and Bilgel, 2008; Backhaus et al., 2020). At the same time, it is becoming increasingly apparent that university teachers face similar experiences, as indicated by, for example, high levels of burnout (Lackritz, 2004), work stress (Gillespie et al., 2001), and low work-life balance (Kinman and Jones, 2008).

Despite university students and teachers both facing compromised well-being, this matter has, to the best of our knowledge, not yet been examined simultaneously within both populations. This is surprising, as numerous studies support the existence of reciprocal relations between aspects of student and teacher well-being in the school context (e.g., Frenzel et al., 2018; Harding et al., 2018), as well as the importance of examining student-teacher relationships in the university context (see Hagenauer and Volet, 2014b). Consequently, more comprehensive research is needed to shed light on opportunities to mutually foster student and teacher well-being in academia.

The current study stems from the positive psychology perspective (i.e., considering well-being in the light of living a well and fulfilled life), which is especially important as research thus far tends to focus on the negative side of well-being in academia. For instance, previous studies have primarily investigated factors causing academic distress (e.g., Gillespie et al., 2001; Benbassat, 2014; Deasy et al., 2016). Additionally, consequences of distress in terms of psychological ill-being are often reported, such as burnout (e.g., Lackritz, 2004), and depression or anxiety (e.g., Stallman, 2010; Larcombe et al., 2016; Backhaus et al., 2020). In contrast, positive aspects of student and teacher well-being such as engagement, positive affect, or life satisfaction seem to be less frequently researched, although exceptions exist (e.g., Schaufeli et al., 2002; Tay and Diener, 2011; Stanton et al., 2016; Stupnisky et al., 2019). Placing an emphasis on the positive side could therefore lend important information to support and enhance student and teacher well-being instead of solely curing ill-being.

Taken together, the current study addresses two primary research goals. First, we investigate how both students and teachers perceive well-being in academia; second, we explore how their well-being might interact. To examine these aspects, we conducted semi-structured interviews with students and teachers regarding their views on and experiences with well-being at the university.

### Theory Surrounding the Conceptualization and Interaction of Well-Being

Well-being constitutes a concept widely used in its various forms and interpretations. These range from an interplay of life satisfaction, absence of negative, and presence of positive emotions (Beiser, 1974), or mastery experiences and personal growth (Tay and Diener, 2011), to a state of optimal experience and functioning (Ryan and Deci, 2001). Still, no generally accepted definition of well-being exists in the literature, which is also reflected in empirical studies. For scientific research, this lack in definition clarity poses a problem, as various theories have been developed throughout the literature and results are consequently difficult to interpret and compare. From a practical perspective, it is also problematic to specify interventions and measures aimed at enhancing well-being without a coherent understanding of the concept. Moreover, in the case of well-being in academia, it seems even more challenging to conclude one final definition (Fraillon, 2004; Centre for Education Statistics and Evaluation, 2015). Therefore, in the present study, we are interested in gaining a comprehensive understanding of how students and teachers themselves define and perceive well-being at the university. Furthermore, gathering information about how they perceive their well-being to interact could provide an even clearer picture of well-being in academia.

Within this process of defining well-being, we use three well-established theoretical perspectives surrounding the conceptualization of well-being to guide our research. First, we consider well-being from a positive psychology perspective. Second, we incorporate the concept of resilience into our research, which is strongly intertwined with well-being (Mguni et al., 2012). Lastly, we consider well-being as a multifaceted construct including certain basic needs which need to be satisfied to ensure well-being. Moreover, when investigating the interactions between teachers’ and students’ well-being, the systemic approach serves as a theoretical basis.

To elaborate, the first aspect aligns with the World Health Organization’s (WHO) view on health, which depicts ‘a state of complete physical, mental, and social well-being and not merely the absence of disease or infirmity’ (World Health Organization [WHO], 2020). This definition supports the positive psychology approach. Based on the salutogenesis concept (Antonovsky, 1987), positive psychology contrasts prior health and psychological world views (Seligman and Csikszentmihalyi, 2000; Azar, 2011). Well-being has previously been viewed as fighting diseases to bring patients to a level of not being ill, and thus, mainly psychological problems such as anxiety disorders, depression, or psychosis were researched. To this day, investigating human illness instead of human health continues, also throughout educational psychology research when examining student and teacher well-being. At the same time, it has become evident that the positive psychology movement, which aims to define well-being as flourishing, positive affect, and engagement, is also important to research (Gable and Haidt, 2005).

Secondly, well-being appears to go hand in hand with the concept of resilience in that it acts as an indicator of well-being (Centers for Disease Control and Prevention [CDC], 2018). However, comparable to well-being, resilience is not clearly defined (Britt et al., 2016). In a broad sense, it relates to an individual’s ability to face negative experiences and activate personal resources to bounce back to the original psychological state prior to the stressor having emerged. This process can, in turn, lead to positive adaptation and psychological growth (Masten, 2001; Tugade and Fredrickson, 2004). Conclusively, building personal resources can be considered a central process when striving for enhanced resilience and well-being (Gable and Haidt, 2005).

Thirdly, well-being depicts a multifaceted construct (Forgeard et al., 2011), including aspects such as physical, social, psychological, and emotional well-being as well as life satisfaction and work engagement (Centers for Disease Control and Prevention [CDC], 2018; World Health Organization [WHO], 2020). Reflecting this multifaceted view, Self-Determination Theory (SDT; Deci and Ryan, 1985; Ryan and Deci, 2000) posits that well-being consists of the satisfaction of three basic psychological needs (BPN): autonomy (the need to experience behavior as self-directed), competence (the need to experience behavior as effectively enacted), and relatedness (the need to interact, be connected to, and care for others). Recent research has addressed the relationship between these needs and enhanced well-being in the academic context, indicating a connection. For instance, having participatory control and flexibility within studies contributes to university students’ satisfaction with their learning environment as well as their feeling of optimal challenge (Stanton et al., 2016). This finding could relate to the satisfaction of the need for autonomy. Moreover, perceived competence has been found to mediate the relationship between university environmental factors (e.g., administrative and research support) and aspects of university teacher well-being (Larson et al., 2017; Stupnisky et al., 2017). Regarding relatedness, the relevance of student-teacher relationships as beneficial factors in both students and teachers has also been identified within the literature (Hagenauer and Volet, 2014b). Taken together, these findings emphasize that the fulfillment of BPN is strongly intertwined with well-being in academia and is worth investigating further.

Lastly, to investigate expected interaction effects between students’ and teachers’ well-being, the systemic approach, based on social constructivism (Burr, 2003), is a fitting epistemological approach. According to social constructivism, social reality is constructed through individual perceptions and interactions with one’s surroundings. Consequently, individuals act and exist within their systems, such as their families or their workplace. However, if a system member displays any emotional, behavioral, or psychological problems, the individual is seen as a symptom carrier of a malfunctioning system or relationship (Minuchin et al., 1978; von Schlippe and Schweitzer, 2015). Translated into the academic context, this implies that a high number of students or teachers, respectively, experiencing psychological distress may not indicate an individual problem of either party. Instead, the issue concerns the relationships between both or even within the academic system as a whole. Thus, it appears insufficient to concentrate well-being interventions solely on students or teachers, but instead, a focus should be placed on the dynamic interplay between both groups.

### Student Well-Being

As previously mentioned, university students frequently demonstrate impaired mental well-being. For students, psychological problems are more elevated compared to the general population in the same age group (Stallman, 2010; Benbassat, 2014; Larcombe et al., 2016). Furthermore, the psychological strain that university students perceive increases after entering university and never returns to the pre-registered level (Cooke et al., 2006; Bewick et al., 2010). These insights seem critical, given that psychological illnesses first emerge before the age of 25 (Veness, 2016). Moreover, on a subclinical level, academic distress can be problematic in that high levels of stress appear to be associated with lower academic achievement (Stallman, 2010), an unhealthy lifestyle (McEwen, 2008), and cognitive as well as behavioral problems in the educational context (Baik et al., 2017). Therefore, the study years depict a potentially sensitive period in an individual’s life (Compas et al., 1986).

From an academic perspective, students’ mental health seems to be of crucial relevance, in a negative, but also especially in a positive manner. Psychological distress impairs academic performance (Stallman, 2010; Deasy et al., 2016), whereas university engagement increases it (Schaufeli et al., 2002). Likewise, positive moods and emotions such as happiness seem to enhance several learning outcomes (Panger et al., 2014; Stanton et al., 2016). Examples thereof include creativity (Baas et al., 2008; Davis, 2009), productivity (de Neve et al., 2013; Oswald et al., 2015) and various cognitive variables important for academic achievement (Fredrickson and Branigan, 2005; Panger et al., 2014). Despite these findings, however, students’ perceptions of well-being and its positive outcomes remain largely uninvestigated (Stanton et al., 2016). Thus, focusing on the positive side of student well-being constitutes a promising research avenue.

### Teacher Well-Being

University teachers hold numerous responsibilities in their role, including teaching students, conducting and publishing research, as well as completing administrative tasks. When combined with additional characteristics of the profession such as working significant overtime hours (Fontinha et al., 2019) and having fixed-term employment contracts (Higher Education Statistics Agency, 2018), it is not surprising that university teachers are considered at-risk for compromised well-being (Kinman and Johnson, 2019). This has also been recognized by, among others, the German Research Foundation, the Dutch Research Council, the Royal Netherlands Academy of Arts and Science, and the Association of Universities in the Netherlands (Rijksoverheid, 2019; Nederlands Wetenschappelijk Onderzoek [NWO], 2020). These organizations acknowledge the high pressure on university teachers in the academic system and seek to support them.

Similar to student well-being, a large body of literature also examines university teacher well-being and aspects that contribute to it (see special issues of Kinman and Johnson, 2019; Mendzheritskaya and Hansen, 2019; Daumiller et al., 2020). Although some studies within this field have found that university teachers report moderate to high levels of job satisfaction (e.g., Kinman and Jones, 2008; Shin and Jung, 2013), most also reflect high levels of burnout and stress (Winefield et al., 2008; Watts and Robertson, 2011; Padilla and Thompson, 2016; Guthrie et al., 2017). Furthermore, university teachers experience additional stressors such as role conflicts and role ambiguity (Richards and Levesque-Bristol, 2016), along with difficulties maintaining work-life balance (Kinman and Jones, 2008; Flaxman et al., 2012; Hogan et al., 2016).

Notably, few studies have explicitly investigated university instructor well-being in a positive light. To acknowledge this research gap and support mechanisms to enhance teachers’ well-being, we aim to explore the concept of well-being from a positive, encompassing, and personal perspective. Some literature on university teachers already suggests the importance of the positive perspective and emphasizes protective and flourishing characteristics. Examples include positive work attitudes (Mudrak et al., 2018), positive emotions for teaching and research (e.g., Stupnisky et al., 2019; Rinas et al., 2020), and positive student-teacher interactions (Hagenauer and Volet, 2014b).

### Interaction Between Student and Teacher Well-Being

Understanding the linkages between student and teacher well-being is important in gaining a comprehensive picture of their functioning in academia. Prior research, primarily in the school context, has supported this point. Regarding emotional well-being, student-teacher relationships have been positively associated with school teacher enjoyment and negatively associated with their anxiety (Hagenauer et al., 2015). Moreover, school teacher and student enjoyment seem to be positively connected (Frenzel et al., 2009), also in a reciprocal manner (Frenzel et al., 2018). Lastly, a link has also been found between school teachers’ instructional characteristics and students’ emotional well-being (Frenzel et al., 2009; Lazarides and Buchholz, 2019). In terms of psychological well-being, higher school teacher well-being has been associated with higher student well-being and lower student psychological difficulties. Reciprocally, lower teacher depressive symptoms have also been associated with higher student well-being (Harding et al., 2018).

Aside from the school context, the importance of the student-teacher interaction may also extend to the university context. Due to systemic differences between schools and universities (e.g., less contact between teachers and students, stronger motivations and autonomy), these findings from the school context cannot be readily transferred to the university context, but require specific investigation (see Daumiller et al., 2016). However, the first few studies that have considered student-teacher interactions in the university context suggest this to be a promising avenue. For example, Hagenauer and Volet (2014a, b), examined the importance of student-teacher interactions as well as university teachers’ emotions, respectively. Regarding the former study, they conducted a systematic review highlighting the importance of the student-teacher relationship in the university context and suggested beneficial effects for students and teachers, also in terms of aspects of well-being. Next, in a longitudinal interview study, they found that student engagement influenced university teachers’ emotions and impacted how they performed in their teaching. Taken together, these findings indicate that the student-teacher interaction warrants further research attention in academia.

### Research Questions

Considering the lack of consistency within the definitions of student and teacher well-being described in the literature, our first research aim was to investigate this conceptualization from a qualitative and positive perspective. To this end, we considered it essential to reflect both students’ and teachers’ thoughts about well-being in order to gain theoretical and practical insights contributing to a clearer definition of the construct within academia. Consequently, the first research aim was formulated by the following questions:

RQ1. a. How do students and teachers conceptualize well-being at the university?
b. How do students and teachers perceive well-being at the university?

The second research aim addressed the knowledge gap surrounding the interaction between student and teacher well-being. Based on literature describing how student-teacher interactions impact certain emotional and psychological aspects of well-being in the academic context, we expected reciprocal relations. In other words, we expected students to mention that teachers were interconnected with their well-being, and vice-versa. It seems relevant to understand the underlying mechanisms of this interaction to gain clearer insights about how to enhance both student and teacher well-being in academia. Thus, our second research focus entailed the following questions:

RQ2 a. What are the direct associations between student and teacher well-being?
b. How do factors contributing to student and teacher well-being relate?

## Materials and Methods

To investigate the research questions above, we conducted semi-structured interviews regarding the conceptualization, perception, and interaction of student and teacher well-being. During the process of conducting these interviews, the study was pre-registered through Open Science Framework^1^.

### Participants

To allow for a broad perspective on the topic and the inclusion of a range of well-being experiences, both the student and the teacher samples were heterogeneous in terms of age, gender, faculty, study or teaching experience, as well as the country in which the participants were from. Specifically, sixteen participants (six students between the ages of 22–29 and ten teachers between the ages of 26–57) from three public universities, one in the Netherlands and two in Germany (n_Dutch_ = 11, n_German1_ = 3, n_German2_ = 2), took part in the present interview study. The participants worked and studied at various faculties, including Education, Economics and Business, Behavioral and Social Sciences, Spatial Sciences, Arts, and the University Medical Center. Moreover, students had between 1 and 4 years of study experience, while teachers had between 1 and 32 years of teaching experience. The student sample additionally contained 50% international students, while the teacher sample contained 42% international teachers.

### Interviews and Procedure

As previously mentioned, to gather an in-depth understanding of university students’ and teachers’ well-being experiences, we conducted semi-structured interviews. Permission to conduct the study was granted by an ethics committee and all participants provided informed consent prior to participating. We first issued a short demographics questionnaire, and then proceeded with the comprehensive, semi-structured interviews. These interviews ranged from 55 to 100 min, were recorded, and then transcribed verbatim. In this process, any personally identifiable information was pseudonymized.

The interviews were conducted by two interviewers in a conversational style, and each interviewer used an interview guide to structure the interviews. The interview guide contained all questions and prompts to be asked, which slightly differed for students and teachers (see additional material^2^). In general, the interview guide consisted of three parts. First, the questions focused on how student well-being is perceived and defined by the participants. Following this, the questions concentrated on teacher well-being, while the last questions concerned the interaction between teachers’ and students’ well-being in academia. A summary of the transcription was sent back to the participants so that they could report if any aspects were misunderstood and further decide to entirely or partly withdraw from the study.

### Data Analysis

To answer the first research question, three phases of coding were implemented. First, the transcribed interviews were initially coded by two expert coders using the values coding approach (Miles et al., 2014). This method states that text fragments are coded depending on whether participants expressed a value, an attitude, or a belief. Based on these initial codes^3^, the two coders proceeded to analyze the data according to thematic analysis as discussed by Braun and Clarke (2006). In line with this method, thematic patterns emerging from the data are identified based on an initial coding phase and then analyzed (see also Ryan and Bernard, 2000). Therefore, the two coders first looked for commonalities across several interviews to identify common codes, which resulted in a preliminary codebook as a guide for coding the remaining interviews. Through this, the codebook was enlarged with new emerging codes until saturation [i.e., the point at which no new information or themes were observed in the data; see Guest et al. (2006)]. Here, it must be noted that it was possible to apply several codes to one passage. For example, a passage coded as ‘belonging’ could simultaneously be coded as ‘social well-being,’ as indicated in the following quote from a university student: “But also, I feel like social is a big thing, like, feeling like you connect with your classmates and instructors.” In the third phase of coding, the final set of codes served for the coding of the same data set again to check whether any initial codes had been missed or needed adjustment. This third phase acknowledges the iterative process within thematic analysis, which requires a constant back and forth processing of the data (Braun and Clarke, 2006). The two final coding phases were conducted using the program ATLAS.ti Scientific Software Development GmbH (designed for qualitative research and data analysis) and performed by the two coders independently to ensure inter-rater reliability. Following this, clusters and themes based on the codes were identified through discussion by the two coders as well as a panel consisting of three additional experts in the field. These themes could emerge both inductively from the data as well as deductively from theory (Hayes, 1997, 2000). The coders additionally worked independently from the aforementioned theoretical framework to ensure both perspectives (Braun and Clarke, 2006).

The second research question was analyzed using a slightly different procedure, yet remained rooted in thematic analysis (Braun and Clarke, 2006) and thus, aimed to find common themes emerging from the data. First, using the same approach and software as in the first research question, the two coders coded the interviews, marking any passages that referred to the student-teacher interaction. It was possible to code a passage as ‘student-teacher interaction’ even if it had already been given a code throughout analyzing the first research question. After this, the passages coded as the ‘student-teacher interaction’ were exported and independently analyzed by the coders with the goal of identifying clusters based on the co-occurring codes, along with searching for new overarching themes. Finally, the coders’ independent findings and the resulting clusters were discussed amongst themselves as well as with the panel of experts, again, to ensure inter-rater reliability. As with the first research question, these clusters and themes could emerge both through inductive or deductive processes.

## Results

### RQ1a. Conceptualization of Well-Being

**FIGURE 1:**
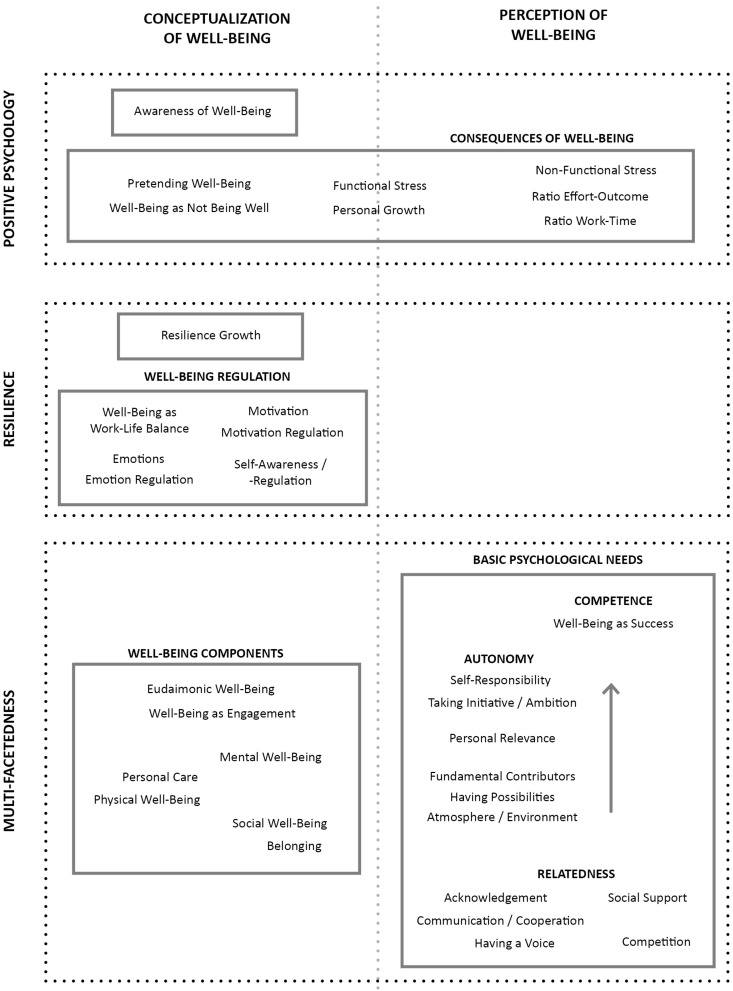
Clustering of codes related to RQ1. This figure includes the codes that emerged during the coding process, which were divided into two main headers: the conceptualization and the perception of well-being, divided by the dotted gray line. The dotted boxes refer to the theoretical facets outlined in the introduction; the other boxes illustrate themes; within these, clusters comprising different codes are displayed. Some aspects overlapped with both the conceptualization and the perception of well-being, and were therefore placed at the intersection between both topics.

An overview of our findings can be found in Figure 1 below. First, two clusters could be seen in the light of positive psychology, namely the awareness of well-being and the consequences of the presence or absence of well-being, respectively. Secondly, resilience growth was described, especially related to well-being regulation. Lastly, some codes emerged that defined well-being in its basic components; emphasizing the multifaceted nature of the concept. We additionally found three clusters concerning the BPN for competence, autonomy, and relatedness, which related rather to the perception of well-being than to its conceptualization.

#### Positive Psychology Approach of Well-Being

##### Awareness of well-being

The ‘awareness of well-being’ code, illustrating a cluster in itself, referred to the presence or absence of an understanding that well-being exists in the academic context and was mentioned by both students and teachers. Regarding the presence of awareness, some students highlighted well-being as a “hot topic” and as “being on the rise so much globally.” Concerning the absence of awareness, in contrast, most students commented on not being aware of either the topic nor offers focused on improving well-being at the university. For instance, when asked about effective coping, one student stated that “it’s not something I think about daily.” Teachers remarked both extremes as well, with some stating that well-being was a topic that they were interested in and aware of, and some, in contrast, mentioning not having thought about the topic before the interview.

##### Consequences of well-being: functional stress and personal growth

The ‘consequences of well-being’ theme focused on the outcomes of the absence or presence of well-being, respectively. Three clusters could be allocated to this theme: ‘pretending well-being,’ ‘functional,’ and ‘non-functional stress.’ In the following section, we solely report on functional stress, relating to the positive outcomes of academic stress and the scope of our study goals. However, the remaining two codes are defined within the codebook and can be found in the additional material.

Functional stress referred to how stress can be perceived in a positive light, as flow, and as an opportunity to develop new competences and self-confidence. For example, teachers stated that being stressed helped them to remain focused and to develop themselves within the academic context. In terms of students, one teacher argued that “part of studying in general is facing problems and that’s a skill you learn during the studies.” Likewise, students themselves mentioned that the experience of stress was an opportunity for them to grow. This “good level of stress” (university student) was intertwined with experiencing study success despite being stressed. Consequently, experiencing stress as “challenging” can be considered functional, and may additionally foster creativity and innovation, as well as personal growth. The ‘personal growth’ code related to learning processes resulting from the experience of functional stress, and included obtaining competences and maturing, but also knowing how to act professionally despite being stressed. It seemed satisfying for teachers to witness this process and see their students successfully developing into skilled academics and adults who can use their personal potential to face future academic stressors.

#### The Resilience Approach of Well-Being

##### Resilience growth

Next to growing personally, functional stress can also result in people growing in terms of becoming more resilient toward stress. This so-called ‘resilience growth’ relates to internal growth not only despite but also due to having experienced negative situations. Teachers were aware of such processes, and admitted that students “have to learn it by doing! That’s also experience. You’ll have to cross it and bounce back and be very tired and very, like, exhausted.” Students mentioned that this growth mechanism led to heightened coping competences due to negative and stressful experiences as well. One student, for instance, phrased her resilience growth in the following way:

[…] one thing that made me cope better with my well-being was having like, like a few really [obscenity], awful things happen, and […] then getting mentally stronger from that. […] I’ve felt better and happier and stronger because I’ve learned to cope with them and deal with them, and I think that’s quite important as well in well-being. (university student)

##### Well-being regulation

In line with the aforementioned coping strategies being regulatory in nature, participants frequently referred to regulating their well-being by means of work-life balance, self-regulation, motivation regulation, and emotion regulation. ‘Well-being as work-life balance’–keeping balance as well as detaching from work or studies with other engagements outside of academia—was emphasized by teachers and students equally as being crucial for their well-being. Although both groups highlighted the importance of activities outside of academia, for students, this also encompassed investing in development toward their future working-selves. Regarding the latter point, teachers admitted that “for university instructors, it’s easier to take a step back because you have other stuff, but for university students your job is to study, so it’s kind of hard.” In contrast, on the teacher side, although teachers acknowledged the difficulty of keeping boundaries between work and private life, this seemed to be more feasible and important to pay attention to: “So, I have learned to say to my students or my colleagues: ‘Sorry I have not been able to do this.’ Knowing that I had time at the weekend or in the evening to do it” (university teacher).

Secondly, ‘self-awareness’ and ‘self-regulation’ appeared to be essential for regulating well-being. For instance, the interviewees reported learning the “things that’ll make you happy” (university student) as well as “what is causing the stress” (university teacher) and using this knowledge for coping. Moreover, a central insight for students was recognizing that their university outcomes and intelligence were independent from one another. Being aware of this discrepancy substantially contributed toward their feeling of well-being: “I really saw that it was, um, my way of learning and not my intelligence, that was-that was a bit of reassurance for me.” These reflective processes were mentioned frequently, including reflecting on the extent of involvement with students as a teacher or reflecting on personal development as a student. Generally, self-reflection appeared to lead to knowledge of one’s strengths, weaknesses, fears, and needs. This process of becoming aware of oneself seemed to be age-related and a skill learnable throughout adultery.

Next, ‘motivation’ and ‘motivation regulation’ were mentioned as important aspects of well-being regulation; yet, they primarily referred to the students’ experiences. Here, they mentioned motivation to relate to their well-being when they could see themselves potentially succeeding in their studies. Therefore, students acknowledged the teacher’s role in stimulating and sparking motivation for specific subjects. Furthermore, being internally motivated due to developmental processes led to students being more persistent: “I kind of, I didn’t want to fail […] I want to pass and don’t want to quit.” However, students also reported experiencing demotivation, although less frequently. These statements referred to situations of feeling overwhelmed with self-responsibility, having too high of expectations, or feeling bored as a result of the teaching style. Finally, in terms of motivation regulation, students and teachers reported various motivational and cognitive strategies, including prioritizing and organizing, self-affirming talk, as well as creating small successes within the learning process.

Lastly, a cluster surrounding ‘emotions’ and ‘emotion regulation’ emerged. Positive emotions covered emotional states such as appreciation, happiness, enjoyment, or excitement, all of which were mentioned equally often by students and teachers. In contrast, negative emotions ranged from feelings of unimportance, misery, and frustration, to anxiety and fear. These negative feelings were generally less frequently mentioned. Yet, primarily students reported them and mainly referred to experiences of failing, because “it never feels good to fail in anything, right?” (university student). Further on, emotion regulation referred to strategies used to regulate their negative emotional states. Examples of these strategies were similar for students and teachers and included exercising, paying attention to one’s lifestyle habits, as well as asking for help and social support. Moreover, most interviewees mentioned rationalizing one’s feelings as an important strategy, as illustrated in the following quote:

I think about that nothing of this is really (p) life-threatening, for example, because these are also really small things, but they seem so big at the moment, and you think, you can’t cope with anything anymore, but if you calm down and think rationally, then you realize that it’s not life-threatening and then you-that you can (p) still do it. (university student)

#### The Multifaceted Nature of Well-Being

##### Well-being components

The multifaceted nature of well-being was illustrated by the ‘components of well-being’ theme, covering clusters of physical, mental, social, and eudaimonic well-being. Concerning ‘physical well-being,’ a considerable number of interviewees reported ‘personal care’ to constitute a central aspect. This care included how they looked after their bodies, such as being attentive toward sleep, nutrition, and exercise. To illustrate a contrasting example, one teacher commented on students during the exam period as “running around the library as ghosts. They didn’t sleep, […] and when they have a total overload with work, you see that physically.”

Next, ‘mental well-being’ appeared to be equally important for students and teachers. This component, however, was mainly mentioned in a negative manner when reporting corresponding experiences. For instance, students reported a lack of mental well-being as a reason to either reach out for professional help or to consider quitting their studies. Furthermore, teachers reported experiences with students who faced mental illnesses as well, such as students becoming thinner in class or student assistants struggling with burnout.

The third component, ‘eudaimonic well-being and engagement,’ referred to perceiving studying as fulfillment or being particularly engaged in study topics. Both appeared to be intertwined, as nearly every third statement coded as eudaimonic well-being co-occurred with well-being as engagement. Concerning engagement, the participants mainly described actively participating in and outside of class, wanting to contribute, as well as actively asking for feedback or further input. Moreover, engagement also seemed to be a central aspect of teachers’ well-being, as expressed by a teacher who described how connecting to the students helped him to engage more with his teaching role. Similarly, eudaimonic well-being illustrated studying or teaching, respectively, as self-realization and meaningfulness, which, in turn, related to personal growth along with intrinsic motivation. For example, one student stressed that it is “nice to, to get good grades and to pass exams and get some, um, feeling of fulfillment, or achievement.” For teachers, eudaimonic well-being also seemed to be important for their well-being and something they value in their jobs:

[…] I like to invent my own things and create my own job and I do like to-to read and be intellectual. That’s just what I like. There’s nothing else I can. (laughing) That is what I have. And I do fit-my brain fits in, with how things work here, because I constantly have ideas and I-so. I never get bored. (university teacher)

Finally, it seemed crucial for students to feel a sense of ‘belonging’ to their academic institution and the people within it, which contributed to their sense of ‘social well-being.’ The latter was described by a student as “how your social life affects you.” This social life included social relationships with friends and family, but also close working connections with fellow students or colleagues. Furthermore, the participants discussed social well-being in terms of taking care of each other, enjoying a socially positive atmosphere, and sharing their concerns. This social connection constituted an aspect that both students and teachers consistently emphasized throughout the interviews. Beyond that, the sense of belonging was just as important, namely, feeling recognized as part of the community and feeling “in the right place” at university (university student). Again, this aspect applied equally to students and teachers, as both groups should ideally “feel being part of the same community” (university teacher). For instance, one teacher stated:

If you are feeling lonely and you-and a lot of students actually move to the city and try-try to start living here apart from their family. So, a lot of students feel disconnected, huh? Um, and have to find a new home or have to define a new home. (university teacher)

### RQ1b. Perception of Well-Being

#### Basic Psychological Needs

Although the clusters of the three BPN, ‘competence,’ ‘autonomy,’ and ‘relatedness’ were not part of the theoretical conceptualization of well-being, we included them nonetheless as contributing factors. Within the interviews, they were specifically mentioned within as well as outside of the student-teacher interaction (see Table 1). In the following sections, we will shortly outline those mentioned outside of the interaction; those mentioned within are reported in the section focusing on the student-teacher interaction (for more details see additional material).

**TABLE 1:** Codes allocated to the basic psychological needs in RQ1 and RQ2.

	Basic psychological need for		
	Competence	Autonomy	Relatedness
Outside student-teacher interaction (RQ.1)	Well-being as success	Self-responsibility Taking initiative/ambition Personal relevance Fundamental contributors Atmosphere/environment Having possibilities	Social support Acknowledgment Communication/cooperation Having a voice Competition
Within student-teacher interaction (RQ.2)	Having control/transparency Structure Feedback	Choice/freedom Flexibility Attention to individual Individual well-being	Course size Support Student-teacher interaction

##### Competence

The need for competence referred to the belief of being able to achieve something and being of value to one’s academic community, which was mainly experienced by students. It appeared that these competence beliefs were highly related to positive feedback from others—both inside and outside of academia. Therefore, experiences of ‘success,’ another code within the competence cluster, strengthened one’s feeling of competence and resulted in a range of positive experiences, such as feelings of pride and motivation. Furthermore, the participants did not only equate success with their well-being, but also vice versa. This is why changes in students’ study outcomes seemed to depict an indicator of their well-being, as one teacher remarked when comparing students who are well to those who are not: “If someone used to be very good and then suddenly there’s a change and he’s delivering bad stuff, then you know something’s wrong.”

Experiencing a lack of competence primarily referred to not knowing how to deal with unfamiliar tasks. Incidences of failing further reinforced these feelings of incompetence. Again, most statements applied to students experiencing and teachers acknowledging these insecurities. Yet, also teachers reported stress due to not knowing how to teach: “[…] when I started at the university as a teacher I was struggling a lot because I didn’t have any teaching experience […] and I didn’t know how to do that.” This perceived incompetence could result in stress, passiveness, and imbalance.

##### Autonomy

A foundation for autonomy in academia comprised creating a certain ‘atmosphere’ and ‘environment,’ ‘having possibilities,’ and satisfying ‘fundamental contributors to quality of life.’ The latter comprised, among others, having adequate housing, financial security, as well as an appropriate office space. As an example, the following quote illustrated the general insecurity tied to being a university teacher: “Academia is a hard life. You get only temporary contracts, you have to earn your own money constantly, you have to constantly show yourself and be innovative-(sighs) crazy culture.” (university teacher).

‘Personal relevance’ seemed to link the basis and the peak of academic autonomy, reflecting the feeling that what one was doing was personally meaningful and mattered. For example, having the impression that studies, or work in the case of teachers, contributed to one’s future could evoke the feeling of personal relevance. Accordingly, one student stated: “And I found it here to be more exciting, to be more (p) um, applicable […] I can do something with it, you know?” Not seeing the purpose of something, in contrast, led to lower motivation and persistence. However, this link applied primarily to the university students compared to teachers.

Finally, ‘taking initiative’ referred to participants’ being ambitious, proactive, and autonomous, hence, taking certain matters into their own hands. Regarding this, teachers aimed to encourage students to take initiative, as illustrated with the following quote:

[The students] should be the ones driving it forward and I should be the one who’s trying to push them to get there. Um, so that requires me sometimes, to, purposely full, take a step back—to let them take the, the, yeah, the steer. (university teacher)

As seen in the previous quote, teachers suggested that by giving students control over what they are doing, students could develop a sense of ‘self-responsibility.’ This, in turn, was defined by being responsible for both one’s well-being as well as one’s studies or work, complementing freedom and autonomy in academia. For instance, one teacher highlighted autonomy in a classroom, telling his students: “Well that’s your responsibility I give you autonomy, but then it’s your responsibility as well. What do you think? And come forward with your conclusions.”

##### Relatedness

When asked about who was responsible for student well-being, one teacher stated: “I’m responsible for my life. But I can use support from others of course.” This quote relates to the final BPN of relatedness, which was strongly associated with the aspect of ‘social support.’ Social support members included a range of social relationships, such as colleagues, friends, and family, or even one’s pet. The ways in which these support members provided social support to others were broad as well, including encouraging them to share, listening to their problems, as well as giving advice, motivation, and confidence.

As illustrated in Table 1, social support was described through three different aspects. ‘Acknowledgment’ concerned situations in which the counterpart acknowledged one’s time and effort, and also respected them as a person, teacher, or student. Moreover, teachers also needed this recognition from their superiors: “You want to feel like your work is valued and that you’re treated with respect” (university teacher). Besides that, ‘communication and collaboration,’ referred to how people in academia communicated with each other. Lastly, ‘having a voice’ addressed being able to voice one’s opinions. Here, however, both students and teachers expressed restrictions in having a voice. To elaborate, students occasionally felt powerless in academic decisions and needed to use their professor’s help to achieve their goal at the university. Teachers, however, criticized that their opinions were not considered in a serious way in faculty matters.

‘Lack of social support’ was experienced when one’s efforts weren’t recognized, for example by neglecting the fact that working in academia encompasses more than attending or giving classes. Yet, also within the academic context, social support was vital and if lacking, led to adverse consequences. This issue was acknowledged by the participants as well: “If you don’t have this basic feeling of being supported and that you have a positive feeling about how things are going; how can we expect you to learn? How can we expect you to teach? Perform research?” (university teacher). Beyond that, a lack in social support was also apparent through various behavioral outcomes, one of which appeared to be ‘competition.’ Specifically, whereas students were struggling with social comparison, teachers competed for funding and promotion opportunities.

### RQ2a. What Are the Direct Associations Between Student and Teacher Well-Being?

**FIGURE 2:**
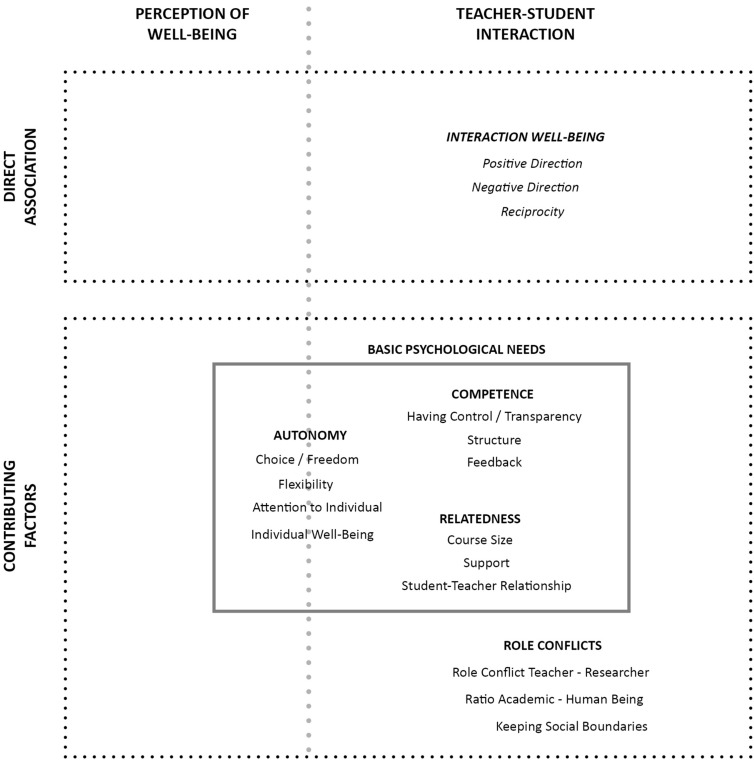
Clustering of codes related to RQ2. This figure includes codes that emerged during the coding process, enriched with overarching themes found while analyzing the interviews for the interaction between students and teachers (RQ.2). These are allocated to the two different aspects of the perception of well-being and the student-teacher interaction, divided by the dotted gray line. The dotted boxes refer to the theoretical facets outlined in the introduction; the other boxes illustrate themes; within these, clusters comprising different codes are displayed. Some aspects seemed to overlap and were therefore placed at the intersection between both overarching topics.

To gain a more integrative picture of well-being in academia, we additionally wanted to understand how student and teacher well-being directly interact. Our interviews indicated that student and teacher well-being were not only “tied together very intimately” (university teacher), but also that this interaction was meaningful and prevalent. This interplay was described in terms of a positive direction, a negative direction, and a reciprocal feedback loop, as will be explained in the following sections^4^. A visual overview of the aspects tied to this research question can be found in Figure 2.

#### Positive Direction

One frequently mentioned overlap consisted of teachers feeling that their well-being was reflected by their lesson planning and teaching practices, and thereby impacted their students’ well-being. For lesson planning, when teachers felt well, this enabled them to prepare “a well-prepared lecture or class, and that’s going to be more satisfying for the students.” Moreover, they also described feeling “more energetic and present” as well as “more interested” when teaching their lessons, which they believed positively impacted their students’ well-being.

Another way in which teachers felt that their well-being positively impacted their students’ well-being appeared to be through direct interpersonal relations. Teachers stated that when they felt well, they experienced stronger and deeper relational ties with their students. One teacher, for instance, explained that she could not be a happy teacher if she taught her students as though she “doesn’t care about them.” This positive interaction was also described in terms of providing support and a warm environment to students such as “listening” or “being there,” which they felt responsible for.

Other times, the positive direction of the interaction was elaborated on in a more general sense. That is, some teachers explained that when they felt well, they were “in a better place” and that there would then be “a high correlation to the students’ well-being.” This perception was strongly reflected in the following quote:

[…] if we as instructors feel supported, taken care of, that everything we’re doing is manageable, that puts us in a position to do our jobs better. And if we can do our jobs better, then that means that we are supporting students in the way that they need and helping them to feel like everything is manageable. So, it’s kind of in that way a ripple effect. (university teacher)

#### Negative Direction

In contrast to the positive side of the interaction, when teachers felt unwell, this seemed to negatively impact their students’ well-being. Examples included feeling overwhelmed, stressed, or having “a bad day,” which seemed to be associated with the interviewees perceiving their lectures to be of lower quality, as well as feeling less connected to students, and having negative emotional experiences within the classroom. In this light, one teacher explained that “if the instructor is stressed or not well prepared or overworked, the students are not going to learn as effectively, which is part of their well-being.”

Similarly, if teachers perceived that their students were not feeling well, this seemed to negatively impact their well-being. On one hand, this was described to occur in the classroom context, where if students were bored, weren’t communicating, or were disengaged, teachers tended to experience negative emotions. On the other hand, they also described having more general feelings of anxiety or worriedness about students who felt unwell, which could be taken home with them and integrated into their personal thoughts:

[…] when they trust you, they tend to give you more information than you actually want. And for me that’s actually bad because I’m very worried and then I want to help and I want to make things good for them. (university teacher)

#### Reciprocity

Aside from single-direction interactions, it was also often mentioned that reciprocity existed between student and teacher well-being. When students seemed well, for example, by interconnecting with teachers or fellow students, participating in exercises, being engaged, or acting friendly and smiling, this positively impacted teachers’ well-being: “That definitely boosts my well-being, like 100%. Knowing that I’ve made a difference, I mean, that’s everything” (university teacher). Their enhanced well-being, in turn, was described as an enabling factor that allowed them to create an atmosphere that promoted student well-being. In contrast, when students explained that they were struggling or appeared to be stressed, withdrawn, or physically unwell, teachers reported having negative emotional experiences. These experiences, in turn, reinforced a negative atmosphere for students, as depicted in the following quote:

[…] if I’m too impacted by their problems then it gets too close to me, it also impacts my well-being and that’s not a good thing. And on the other hand, […] if I’m struggling and if I didn’t sleep and if I’m not relaxed, then I can’t fulfill the expectations that I have for myself as a teacher. (university teacher)

Thus, an important insight to be drawn is that student and teacher well-being appear to be connected through a reciprocal feedback loop. In particular, when one population experienced compromised well-being, this seemed to negatively impact the other population’s well-being, which thereby perpetuated a negative atmosphere. Moreover, when one population felt well, this positively impacted the other population’s well-being, resulting in a positive feedback loop.

### RQ2b. How Do Factors Contributing to Student and Teacher Well-Being Relate?

Aside from the direct interaction between student and teacher well-being, contributing factors also emerged. These factors were not explicitly defined as components of well-being but rather as aspects that influenced student or teacher well-being and pertained to the interaction. To this end, two main themes describing these factors emerged, namely ‘BPN’ and ‘role-conflicts’ (see Figure 2). Regarding the theme of BPN, the clusters of ‘competence,’ ‘autonomy,’ and ‘relatedness’ emerged, which exclusively focused on the role that teachers held in supporting students’ fulfillment of these needs. It is important to note that the present BPN theme differed from the one mentioned in the first research question. Here, the focus was instead on the student-teacher interaction, and thus warranted further distinction (see Table 1 or Figures 1, 2, respectively, for a comparison of the different codes involved in the clusters of BPN).

#### Basic Psychological Needs Connected to the Student-Teacher Interaction

##### Competence

The cluster of competence in terms of the student-teacher interaction emerged through ‘control/transparency,’ ‘structure,’ and ‘feedback.’ Teachers seemed to promote these competence factors and, in turn, supported students’ sense of capability in their studies along with their overall well-being. For example, ‘feedback’ referred to gaining critical information about how one is performing. Specifically, teachers believed students would be more satisfied with and feel more competent in their studies if there were “more opportunities for feedback loops back and forth between instructors and students” (university teacher). This aspect was also mentioned frequently by students and is well-elaborated in the following quote:

[…] you were pushed so hard and then never given feedback. You were just told to […] write your essay every week, and then you’d hand in the essays and then you wouldn’t get them back so you never really know what you had to improve on. (university student)

##### Autonomy

The cluster of autonomy concerning the student-teacher interaction related to ‘choice/freedom,’ ‘flexibility,’ ‘attention to individual,’ and ‘individual well-being’ (see additional material). Generally, teachers seemed to understand the importance of promoting a sense of autonomy in students through these factors which allowed students to feel more independent and ultimately more well. For example, choice/freedom referred to the ability to decide what, when, and how one does something. For students, this freedom appeared to be rather important in terms of feeling that they could complete tasks and assignments in the way they wanted to. One student explained this by stating “it’s up to you, how you-how you do it. And I mean that’s also, a really nice task in order to develop responsibilities and […] to take care of yourself.” Likewise, teachers believed that providing students with choice and freedom in their courses gave students a sense of autonomy which they believed to be “very important for […] students’ success” (university teacher).

##### Relatedness

Lastly, the cluster of relatedness in reference to the student-teacher interaction was formed through the codes of ‘course size’ and ‘support,’ as well as the ‘student-teacher relationship’ (see additional material). Here, teachers supported students’ feelings of social connectedness and their sense of being a part of a caring environment through these factors. Of particular relevance was the student-teacher relationship, which was mentioned in every interview. It appeared that through this relationship, a strong sense of connectedness and cooperation could positively impact both student and teacher well-being. Teachers, for example, described the feeling that they could connect on a deeper level with their students rather than seeing them as student numbers. This point was further elaborated on by a teacher who was asked what could be changed in order for students to be satisfied with their studies:

It would look like the- I enter the building and there are a couple of students and we drink coffee together and […] (p), they have questions and we talk about our ideas about social problems that there are and how different theories apply to that. (university teacher)

#### Role Conflicts Connected to the Student-Teacher Interaction

There were additionally numerous role conflicts described which were intertwined with the student-teacher interaction. This theme could be grouped into the clusters of ‘role conflict teacher–researcher,’ ‘ratio academic–human being,’ and ‘keeping social boundaries.’

The teacher–researcher role conflict was described as the feeling of having difficulties maintaining a high status in both teaching and research, and a tendency to invest more effort into one over the other. Teachers reported that they aspired to be supportive teachers to their students, but that this could be difficult with their simultaneous desire to succeed in research, as implied in the following quote: “And um, it is competing with, of course, research, […] it is taking away time from something else. Just to be the teacher I want to be. And, yeah, that is a bit sad” (university teacher).

The ratio academic–human role conflict referred to teachers thinking of their students as individual people having emotions and needs, rather than solely in an academic sense as ‘students.’ Teachers seemed to realize the importance of treating their students in a more personal and supportive manner. One teacher expressed that students “shouldn’t be a subject, they should be human beings with all their issues. This creates a more human relationship than just grading and saying why you passed or you failed.”

Lastly, keeping social boundaries in terms of the student-teacher interaction constituted the feeling that teachers needed to maintain boundaries concerning their personal connection with students. Specifically, teachers seemed to be personally impacted by students’ problems. While most teachers desired to help their students and to listen, it was also important for them to uphold a “safeguard” to prevent them from lying awake at night thinking “how do we get to this deadline?” (university teacher), as elaborated on in the following quote:

And my big problem is that I tend to be involved too much. Like when someone tells me about a problem or when I see my students suffering or struggling then it comes very close to me. And part of taking care of my well-being is to keep them at a distance. (university teacher)

## Discussion

The overall aim of this paper was to better understand how students and teachers perceive well-being in academia, as well as how their well-being interacts. Within this, theoretically sound well-being perspectives were considered including positive psychology, the BPN, and resilience. To this end, important findings could be drawn concerning both research questions, which incorporated these perspectives. Strengths of the present work include having gathered comprehensive information from interview data, being able to incorporate results based on lived experiences into the literature, and addressing various research gaps such as a lack of positive psychology literature surrounding student and teacher well-being in higher education.

### Conceptualization and Perception of Well-Being in Academia

One of the primary insights gained throughout the process of conceptualizing well-being was the fact that the clusters aligned with our theoretical assumptions of well-being. Therefore, we will focus on the four main findings concerning the conceptualization and perception of well-being: (1) the positive connotation of stress and well-being, (2) the role of resilience and well-being regulation, (3) the multifaceted components that were found to define well-being, as well as (4) the significance of the BPN.

First, it was notable that despite mentioning negative aspects, the participants defined well-being and stress in academia largely in a positive light. For instance, emphasizing awareness of well-being and functional stress as well as personal growth underlines the importance of a positive approach in well-being research. However, experiencing the “right amount of stress” to function properly, as stressed by students and teachers, is not a novel concept: what we identified as functional stress may relate to prior theoretical approaches such as flow theory (Csikszentmihalyi, 1990) or eustress (Selye, 1980). Both theories are intertwined and refer to a state of optimal performance along with a feeling of competence when experiencing a certain degree of stress. These notions are of considerable importance in educational sciences as well, and, thus, also in well-being research in academia (Gibbons, 2015; Mesurado et al., 2016). In detail, flow theory addresses the state of feeling challenged and of being fully immersed in what one is doing, and therefore constitutes an essential notion in positive psychology research (Csikszentmihalyi, 1990). Eustress, defined as positive or good stress and, thus, as the opposite of distress, seems to be beneficial and to relate to various well-being factors (Selye, 1980). Indeed, previous research has linked both eustress and flow to concepts such as engagement, motivation, belonging, as well as competence; aspects that were often found in our data as well (Gibbons, 2015; Mesurado et al., 2016). Hence, stress theories such as flow and eustress seem to conceptually overlap with our definition of functional stress as a positive psychology aspect.

Secondly, the emphasis on resilience growth constituted a relevant finding as well. Although the respondents did not refer to resilience specifically, resilience growth was mentioned throughout various interviews. Given the unlikeliness of participants to directly refer to such an abstract theoretical construct, it seems rather unsurprising that they did not mention resilience itself. According to Mansfield et al. (2016), resilience depicts not only a capacity, but also a process and an outcome. The latter two aspects align with the process of resilience growth, or, in other words, the sustainable outcome of effective coping in situations of distress (Bonanno, 2004; Reich et al., 2010). As the participants explained how adverse experiences formed their capacity to face future stressors, it can be concluded that resilience growth plays a crucial role in well-being in academia as well. To this end, primarily students reported having experienced resilience growth throughout their studies compared to university teachers. Still, resilience appears to be relevant in the workplace as well and might therefore also be essential for university teachers (Bonanno, 2004; Britt et al., 2016; Mansfield et al., 2016). Consequently, this aspect warrants further research attention concerning university teachers.

As a third objective of the study, we wanted to identify the core components for conceptualizing well-being in academia, which emerged from the interviewees’ responses. Acknowledging the multidimensional approach of well-being, we expected various aspects of well-being to arise in our data. In line with this, we found an interplay between mental, physical, social, and eudaimonic well-being. In other words, students and teachers expressed that being well for them referred to being mentally healthy, taking care of themselves physically, feeling that they belonged to their institution, and being engaged in what they were doing. This definition coincides with the WHO’s conceptualization of well-being stated within the introduction (World Health Organization [WHO], 2020), yet is extended by the eudaimonic well-being facet.

Misselbrook (2014) criticizes the strictly positive view of health, such as the one advertised by WHO, to be a “utopian vision” and an “unattainable ideal” (p. 582). Such a positive approach, fixated on a presence of positive well-being aspects, seems to neglect certain aspects of human life, including negative emotions and undesirable experiences. We agree with this critique to a certain extent, seeing the problem of stigmatizing a sense of not feeling well. In fact, we found that several participants appeared hesitant to acknowledge times in their life when they felt unwell and rather pretended to be well (see ‘pretended well-being’ in the codebook). We recognize these dynamics in the various states of well-being, which might impact one’s sense of well-being negatively from time to time. Yet, we believe that human beings can experience personal well-being despite—or in the case of resilience growth, even because of—negative circumstances. In line with this, Misselbrook (2014) conclusively defines well-being, from a medical perspective, as an “unimpaired flourishing” (p. 582). We strongly agree with this view in that individuals are capable of overcoming potential obstacles as well.

Our core conceptualization appeared to be combined with the potential of personal growth due to functional stress as well as with effective regulation of one’s well-being resulting in resilience growth. In this light, related aspects that were not defined as core aspects of the definition were nonetheless strongly intertwined. For instance, aspects such as emotional well-being (Diener et al., 1999), which were not directly reported as well-being components by the participants, played a regulatory or contributing role, respectively. Therefore, these aspects were just as important to include. However, it appeared difficult during the analysis to draw a line between what well-being constituted and which factors solely contributed to a sense of well-being. This is important to keep in mind concerning our decision to define aspects of SDT and well-being regulation as contributing or regulatory factors instead of core elements of well-being. Therefore, given the heterogeneity in conceptualizing and perceiving well-being as well as the difficulties in differentiating contributing factors from core components, other researchers might have come to different conclusions.

Lastly, we postulated that the BPN for competence, autonomy, and relatedness might play an essential role for perceiving well-being in academia. Indeed, all three BPN, autonomy, competence, and relatedness, can be considered as highly relevant when investigating well-being in academia. How autonomous students and teachers feel, how competent they perceive themselves to be, and how related they are to their social surroundings constitute central findings concerning the perception of well-being at the university. Taken together, this assumption reflects on previous research (e.g., Stanton et al., 2016; Larson et al., 2017; Stupnisky et al., 2017; Backhaus et al., 2020) and should also be considered to guide future investigations. On the one hand, all three aspects seemed to be relevant and to contribute to a sense of well-being for students as well as teachers. On the other hand, also within the student-teacher interaction, the satisfaction of these BPN depicted a central aspect. To elaborate, teachers seemed to contribute to their students’ well-being in satisfying their need for competence, autonomy, as well as relatedness when interacting with them. Within this, the need for relatedness concerning the student-teacher relationship, which has been previously suggested (Hagenauer and Volet, 2014b), played a strikingly important aspect of students’ and teachers’ well-being in our data as well. Having already expected such an outcome, the insights gained when investigating this interaction more in-depth were of special interest.

### Insights Concerning the Interaction Between Student and Teacher Well-Being

In addition to the conceptualization and perception of well-being, the present study also shed light on important aspects concerning the until now uninvestigated link between students’ and teachers’ multi-faceted well-being in academia. Firstly, in line with expectations, a strong interaction was found in that when university teachers had better or worse well-being, this was, in their perception, associated with better or worse well-being in their students as well. This interaction appeared to be both multifaceted and multidirectional. Regarding the former, in line with Gasper (2004), who argued the importance of respecting the diversity of well-being, we observed that students and teachers were connected through various aspects of their well-being. In particular, their emotional well-being, social well-being, and mental well-being appeared to be strongly intertwined. This resonates with prior literature where, albeit considering individual facets of well-being, the importance of these reciprocal links has also been suggested (e.g., Hagenauer et al., 2015; Frenzel et al., 2018; Harding et al., 2018). Moreover, regarding the multidirectionality, the presence of reciprocal relations is a key finding and suggests that students and teachers can mutually benefit from positive states of well-being. This coincides with research in the university context which discusses the importance of reciprocal effects of emotional well-being (Hagenauer and Volet, 2014b), as well as the transactional nature of the student-teacher relationship (Cotten and Wilson, 2006).

Aside from the interaction itself, it was found that through various factors, students and teachers could impact one another’s well-being. Although the student-teacher relationship has consistently been recognized as a powerful factor in higher education research (Hagenauer and Volet, 2014b; Kezar and Maxey, 2014), our study extends this finding to the construct of well-being. In particular, teacher-related factors seemed to be an important determinant of student well-being, as also suggested by McCallum and Price (2010), who stated that well teachers promote well students. This falls in line with the systemic approach, where the university can be considered a co-contributing atmosphere in which it is critical to concentrate on the interplay of both groups for optimal well-being. However, as most research focuses on the student side of the equation, our findings suggest that it is important to consider both student and teacher well-being to determine what factors affect these relationships for both groups. Thus, a stronger emphasis should be placed on efforts to simultaneously support both populations.

Adding to this, the role conflicts described by teachers were also an interesting finding that emerged from our data. As mentioned by Hagenauer and Volet (2014b), teachers who primarily self-identify with their researcher role may hold different perceptions and values compared to those who identify more as a lecturer or supervisor. Indeed, empirical research documented that role conflicts between teaching and research and academics’ attitudes regarding the interrelatedness of these domains is tied to aspects of their well-being (Daumiller and Dresel, 2018, 2020). Extending this logic implies that not only switching between teaching and research roles, but also switching between different teacher roles (lecturer and supervisor) might be conflicting. In the present study, we did not restrict the learning environment to either the interaction in class or in a thesis supervision relationship. Thus, it could be of interest to distinguish these two relationships to investigate if they might differ in quality concerning well-being.

### Limitations

Despite the contributions that the present study makes to the literature, several limitations must be borne in mind when interpreting the findings. Firstly, we used a selective sample of university teachers and students and thus, our sample runs the risk of certain biases and causal relations cannot be drawn. Specifically, our sample voluntarily agreed to participate in the study, which likely reflects at least some levels of successful coping at the university in terms of well-being and interest in the topic. Thus, our findings might underestimate ill-being factors at universities; however, due to our positive focus on well-being and its facets, we do not consider this as problematic in terms of distorting our findings. Moreover, although an iterative process is commonly used in qualitative research (Watling and Lingard, 2012; Levitt et al., 2018), it can potentially lead to methodological issues in terms of changes within the interview script. In our case, the explicit question of whether and how the interviewees believed student and teacher well-being were intertwined was problematic and led to exclusively teachers reporting on an explicit interaction. Students, in contrast, tended to explain ways in which teachers supported their well-being indirectly rather than to mention the interaction directly. Finally, our study focused on the comprehensive findings gained from using a qualitative research design, which by nature entails limitations concerning quantitative insights. Thus, future studies could profit from incorporating mixed-method or quantitative designs into this line of research.

### Implications and Future Research

Given that well-being in educational contexts is becoming increasingly important due to heightened stress and burnout levels, as mentioned within the introduction, we aimed to contribute to a coherent definition of well-being in academia for future research. Our conceptualization, including its core components as well as its regulatory mechanisms and contributors, might contribute to finding a common understanding. Such an overall definition of well-being is expected to benefit future research and to help researchers to talk about the same concept when investigating well-being in academia.

Adding to this, further relevant theoretical frameworks could be considered in future research on student and teacher well-being. First, the Job-Demands Resources Model (Bakker and Demerouti, 2007), in which stress and compromised well-being can be considered as an imbalance between demands and resources, may be especially relevant. Both students and teachers mentioned numerous responsibilities and demands that created stress, but also that different aspects that may be considered as personal resources, such as resilience growth or maintaining work-life balance, seemed to help. Second, numerous passages in our interview data reflected the usefulness of Achievement Goal Theory as a lens for investigating well-being, which characterizes motivation as “more or less strong strivings toward task mastery and competence development or toward superiority and competence demonstration” (Daumiller and Dresel, 2020, p.1). Specifically, in describing their well-being experiences, students and teachers seemed to reflect different goal orientations, such as learning goals (being focused on gaining knowledge and competencies), relational goals (being focused on fostering close and caring relationships), appearance avoidance goals (being concerned with appearing incompetent to others), and work avoidance goals (being focused on getting by with little effort).

Despite evidence of interaction effects between students and teachers, there exists far more research on initiatives to promote students’ well-being in academia than university teachers’ well-being (Fernandez et al., 2016). This tendency is also reflected in present strategies to enhance well-being in academia, such as the Australian Health Promoting University Network or various interventions focused on helping the individual to face academic distress (for examples, see Gleeson, 2001; Conley et al., 2015; Dawson et al., 2019). Such academic initiatives to enhance student well-being have also been initiated in the Netherlands and Germany, such as the Dutch National Network Student Well-Being or German awareness-raising initiatives. Despite examples such as the Okanagan Charter, an international charter for promoting health in universities and colleges for both staff as well as university student well-being, similar initiatives remain scarce. Thus, to supplement the student side of well-being in academia, future research as well as university policy should further focus on practical methods to support academics in dealing with compromised well-being. In fact, the interconnectedness and reciprocity between staff and student well-being, which our findings supported as well, emphasize that to enhance mental health, university strategies must focus on both populations (Fernandez et al., 2016). Furthermore, policies should stress factors that are conducive to supporting the student-teacher relationship, as also suggested by Hagenauer and Volet (2014b) as well as Frenzel et al. (2016). Ultimately, positive interactions between students and teachers should be further encouraged as they constitute powerful tools in promoting well-being in academia.

## Conclusion

Taken together, the present study contributes to a better understanding of what well-being means for students and teachers in the university context as well as how their well-being interacts. In particular, our findings indicate that well-being encompasses the core elements of mental, physical, social, and eudaimonic well-being. These core elements were strongly intertwined with several important contributing and regulatory factors, such as the BPN and resilience growth, keeping a work-life balance, as well as emotion, motivation, and self-regulation. Moreover, the aspects within our conceptualization reflected a positive, resilience-based, multifaceted, and basic need fulfillment approach, as outlined in the results section. In line with these theoretical notions, indications of a pronounced and dynamic interplay between student and teacher well-being were found. Concrete next steps may involve quantitatively investigating the aspects found within this paper to further understand their impact on students’ and teachers’ well-being. This approach, in turn, might lead research and practical initiative efforts further in understanding how to promote well-being at the university and foster students’ and teachers’ well-being shoulder-to-shoulder.

## Data Availability Statement

The datasets presented in this article are not readily available because sharing the data openly was not possible due to the sensitivity of the qualitative data. Requests to access the datasets should be directed to l.kiltz@rug.nl.

## Ethics Statement

The studies involving human participants were reviewed and approved by Ethics Committee of the Department Teacher Education, University of Groningen, Netherlands. The patients/participants provided their written informed consent to participate in this study.

## Author Contributions

All authors listed have made a substantial, direct and intellectual contribution to the work, and approved it for publication.

## Conflict of Interest

The authors declare that the research was conducted in the absence of any commercial or financial relationships that could be construed as a potential conflict of interest.

## Footnotes

1 The pre-registration can be found at https://osf.io/2bu7v

2 The additional material is available at https://doi.org/10.34894/Q6DFLB and contains consent forms, the participant information letter, the interview guides, as well as the final codebook which emerged throughout the analysis. The data itself is not included due to the high sensitivity of the topic.

3 For clarification, the term ‘code’ refers to a label that a particular passage in the interview transcription was given, the term ‘cluster’ refers to a grouping of similar codes, and the term ‘theme’ refers to an overarching topic encompassing similar clusters, in line with the definitions of Braun and Clarke (2006) as well as Ryan and Bernard (2000).

4 RQ2a only contains quotes from teachers, as the question of whether and how student and teacher well-being interacted was solely asked to the teachers.
